# SNHG8 Promotes the Progression of Epstein–Barr Virus-Associated Gastric Cancer *via* Sponging miR-512-5p and Targeting TRIM28

**DOI:** 10.3389/fonc.2021.734694

**Published:** 2021-10-15

**Authors:** Changyan Zou, Jinrong Liao, Dan Hu, Ying Su, Huamei Lin, Keyu Lin, Xingguan Luo, Xiongwei Zheng, Lurong Zhang, Tao Huang, Xiandong Lin

**Affiliations:** ^1^ Laboratory of Radiation Oncology and Radiobiology, Fujian Medical University Cancer Hospital and Fujian Cancer Hospital, Fuzhou, China; ^2^ Department of Pathology, Fujian Medical University Cancer Hospital and Fujian Cancer Hospital, Fuzhou, China; ^3^ Department of Genetics, Yale University School of Medicine, New Haven, CT, United States; ^4^ Bio-Med Big Data Center, Chinese Academy of Sciences (CAS) Key Laboratory of Computational Biology, Shanghai Institute of Nutrition and Health, Chinese Academy of Sciences, Shanghai, China; ^5^ Fujian Provincial Key Laboratory of Translational Cancer Medicine, Fuzhou, China

**Keywords:** EBVaGC, SNHG8, miR-512-5p, *TRIM28*, cell proliferation, migration, invasion

## Abstract

SNHG8, a family member of small nucleolar RNA host genes (SNHG), has been reported to act as an oncogene in gastric carcinoma (GC). However, its biological function in Epstein–Barr virus (EBV)-associated gastric cancer (EBVaGC) remains unclear. This study investigated the role of SNHG8 in EBVaGC. Sixty-one cases of EBVaGC, 20 cases of non-EBV-infected gastric cancer (EBVnGC), and relative cell lines were studied for the expression of SNHG8 and BHRF1 (BCL2 homolog reading frame 1) encoded by EBV with Western blot and qRT-PCR assays. The relationship between the expression levels of SNHG8 and the clinical outcome in 61 EBVaGC cases was analyzed. Effects of overexpression or knockdown of *BHRF1*, SNHG8, or *TRIM28* on cell proliferation, migration, invasion, and cell cycle and the related molecules were determined by several assays, including cell proliferation, colony assay, wound healing assay, transwell invasion assay, cell circle with flow cytometry, qRT-PCR, and Western blot for expression levels. The interactions among SNHG8, miR-512-5p, and *TRIM28* were determined with Luciferase reporter assay, RNA immunoprecipitation (RIP), pull-down assays, and Western blot assay. The *in vivo* activity of SNHG8 was assessed with SNHG8 knockdown tumor xenografts in zebrafish. Results demonstrated that the following. (1) *BHRF1* and SNHG8 were overexpressed in EBV-encoded RNA 1-positive EBVaGC tissues and cell lines. *BHRF1* upregulated the expressions of SNHG8 and TRIM28 in AGS. (2) SNHG8 overexpression had a significant correlation with tumor size and vascular tumor thrombus. Patients with high SNHG8 expression had poorer overall survival (OS) compared to those with low SNHG8 expression. (3) SNHG8 overexpression promoted EBVaGC cell proliferation, migration, and invasion *in vitro* and *in vivo*, cell cycle arrested at the G2/M phase *via* the activation of *BCL-2*, *CCND1*, *PCNA*, *PARP1*, *CDH1*, *CDH2 VIM*, and *Snail*. (4) Results of dual-luciferase reporter assay, RNA immunoprecipitation, and pull-down assays indicated that SNHG8 sponged miR-512-5p, which targeted on *TRIM28* and promoted cancer malignant behaviors of EBVaGC cells. Our data suggest that BHRF1 triggered the expression of SNHG8, which sponged miR-512-5p and upregulated *TRIM28* and a set of effectors (such as *BCL-2*, *CCND1*, *CDH1*, *CDH2 Snail*, and VIM) to promote EBVaGC tumorigenesis and invasion. SNHG8 could be an independent prognostic factor for EBVaGC and sever as target for EBVaGC therapy.

## Introduction

Epstein–Barr virus (EBV), belonging to the herpes virus family (1, 2), is a causative virus for many malignancies including nasopharyngeal carcinoma (NPC) and Burkitt lymphoma (3–5). The existence of the EBV genome in gastric cancer (GC) was first detected using a polymerase chain reaction (PCR) in 1990 (5, 6). So far, EBV has been detected in approximately 10% of gastric cancers worldwide (6, 7).

EBVaGC is a distinct subset of GC determined by comprehensive molecular analyses, which has unique clinical pathological features, including *PIK3CA* mutations, DNA hypermethylation, amplification of *JAK2* and *PD-L1*/*PD-L2* (1, 7). However, the underlying molecular mechanism of the development and progression of EBVaGC is unclear.

LncRNAs, non-coding RNAs with their sizes longer than 200 nucleic acids, were initially considered as “junk” or “genomic dark matter” without function (8, 9). However, recently, lncRNAs have been found to participate widely in various physiological and pathological processes, including cancer progression (9–12). Dysregulations of lncRNAs are associated with a variety of cancer malignant behaviors, such as cell migration, invasion, metastasis, gene transcription, and tumorigenesis (13, 14). For example, SNHG8, located on 4q26 and encoding small nucleolar RNAs (snoRNAs), was detected in multiple malignant tumors, including non-small-cell lung cancer, hepatocellular carcinoma (10, 15), and pancreatic adenocarcinoma (11, 16). LncRNAs, as oncogenes or tumor suppressors, are directly involved in tumorigenesis, cell cycle arrest, apoptosis, epithelial to mesenchymal transition (EMT), cell migration, invasion metastasis, and chemoresistance *via* activating the JAK2/STAT3 pathway or Wnt/β-catenin signaling (17–20). Therefore, lncRNAs might be excellent candidates for individualized treatments and monitoring the prognosis of gastric cancer.

We have reported that SNHG8 was a key regulator of EBVaGC by an integrative analysis of lncRNA and mRNA expression (21). This study was to explore the molecular mechanisms of SNHG8 contributing to the progression of EBVaGC *via* sponging miR-512-5p and targeting TRIM28 and a set of effectors.

## Methods

### Collection of Tissue Specimens

After GCs were surgically resected, tissues from 61 patients with EBER-1-positive EBVaGC and 20 patients with EBER-1-negative EBVnGC were identified by *in situ* hybridization (22) and used in this study. All GC tissue adjacent normal tissues were quickly frozen in liquid nitrogen and stored at −80°C until use. The study protocol was approved by the Ethics Committee of the Fujian University Cancer Hospital, Fujian Cancer Hospital (Fuzhou, China), and the written informed consents from all the participators were obtained. All protocols are consistent with the Helsinki declaration.

### *In Situ* Hybridization

EBER1 *in-situ* hybridization (ISH) was carried out with the EBER1 probe ISH kit (ZsBio, Beijing, China) on FFPE (formalin-fixed and paraffin-embedded) tissue slides. The tumor cells with clear nuclear staining of EBER1 were considered EBV positive.

### Cell Lines and Cell Culture

AGS-BX1 [EBV-infected GC cell line (23)] was obtained from Dr. HL Chen (The University of Hong Kong); MKN-28 (GC cell line) and GES-1 (normal epithelial cell line of gastric mucosa) were purchased from ATCC. Cells were cultured in DMEM/F-12 containing 10% (v/v) heat-inactivated fetal bovine serum (FBS) and 100 U/ml of streptomycin/penicillin mixture (Gibco; Thermo Fisher Scientific, Inc., Waltham, MA, USA) in a humidified 37°C incubator with 5% of CO_2_.

### Construction of Overexpression or Knockdown SNHG8 Cell Lines

For the overexpression or knockdown SNHG8 in cell lines, the virus vectors were constructed by Hanheng Biotechnology Co Ltd (Beijing, China). To create cell lines with SNHG8 overexpression, HBLV-SNHG8-OE (pHBLV-CMV-mcs-3flag-EF1-ZsGReen-T2A-PURO inserted with SNHG8 gene) was used. The original vector was used as vector alone control (HBLV-NC). For SNHG8 knockdown in cells, HBLV-SNHG8-shrna1, HBLV-SNHG8-shrna2, and HBLV-SNHG8-shrna3 were used and their parental vector pHBLV-U6-MCS-CMV-Zs/m cherry was used as vector alone control. AGS-BX1 cells cultured in six-well plates(5 × 10^5^/well) were infected with 10 MOI of viral vectors in the presence of 6 μg/ml of polyamine. After 48 h, the cells were selected with 2 μg/ml of puromycin for 2 weeks to obtain stable infected cells. Newly established cells were named as AGS-BX1/NC, AGS-BX1/SNHG8-OE, or AGS-BX1/SNHG8-SH. The symbols -OE and –SH mean overexpression and knockdown, respectively.

### Construction of Overexpression BHRF1 AGS Cell

The plasmids pCDNA3.1-flag-N-humanized-BHRF1 and pCDNA3.1-flag-NC were purchased from Hanheng Biotechnology Co. Ltd. (Beijing, China); AGS cells in six-well plates(5 × 10^5^/well) were transfected with 2 μg plasmid. After 48 h of transfection, the cells with overexpression of BHRF1 or with empty vector were named as AGS/BHRF1-OE or AGS-NC.

### Reverse-Transcription Quantitative Polymerase Chain Reaction

Total RNAs were isolated using the RNeasy Mini Kit (Qiagen, Valencia, CA, USA). To reversely transcribe total RNAs into complementary DNA (cDNA) of SNHG8, hsa-miR-512-5p, *TRIM28*, *BCL-2*, *CCND1*, *PCNA*, *PARP1*, *CDH1*, *CDH2 VIM*, and *Snail* in AGS-BX1 cells, the RevertAid First Strand cDNA Synthesis Kit (Thermo Fisher Scientific; Waltham, MA, USA) and miScript Reverse Transcription Kit were used. The generated cDNAs were used as templates for assessing gene expressions with miScript SYBR Green PCR Kit (Qiagen GmbH, Hilden, Germany) and LightCycler 480 SYBR Green I Master (Roche Applied Science; Indianapolis, IN, USA). The housekeeping gene glyceraldehyde phosphate dehydrogenase (GAPDH) served as the internal control of *TRIM28*, *BCL-2*, *CCND1*, *PCNA*, *PARP1*, *CDH1*, *CDH2 VIM*, and *Snail*, whereas U6 small nuclear RNA served as the internal reference for the expression of hsa-miR-512-5p. Relative gene expression was calculated with the 2^−ΔΔCt^ method.

Relative mRNA expression levels of SNHG8 were quantified in all EBVaGC samples using the comparative 2^−ΔΔCt^ method. *GAPDH* was used to normalize expression levels of SNHG8.

### MTT Assay and Cell Cycle Analysis

Twenty-four hours after infection, the cells were collected, resuspended in the medium, and inoculated into 96-well plates (5,000 cells in 100 μl/well). The cell proliferation was assessed at 24, 48, 72, and 96 h later. At every time point, 20 μl of the MTT solution (Promega; Madison, WI, USA) was added into each well, followed by incubation at 37°C for another 4 h. One hundred fifty microliters of dimethyl sulfoxide was added into each well and shaken for 10 min to fully dissolve the MTT. The absorbance of each well was detected at OD_490_ nm by Model 680 reader (Bio-Rad Laboratories, Inc., Hercules, CA, USA). Cell cycle analysis was done using Muse™ Cell Cycle Kit™ Cell Analyzer (Millipore, USA) in accordance with the manufacturer’s instructions (24).

### Transwell Invasion Assays

The transwell chambers (8 μm diameter; Corning Inc., Corning, NY, USA) were used for cell migration assessment, while the upper chambers precoated with Matrigel (BD Biosciences, USA) were used for the invasion assay. Cells were harvested, washed with PBS, and resuspended in DMEM/F-12 without FBS. One hundred microliters of 2 × 10^4^ cells were added into the upper chambers of a 24-well plate with 500 μl of media. At 24 h after seeding, the cells in the upper chambers were gently removed, and the migrated or invaded cells in the bottom side were fixed with 100% methanol, stained with 0.5% crystal violet, washed with PBS, and observed under inverted microscope (Olympus Corporation, Tokyo, Japan).

### Colony Formation Assays

For colony-formation assay, about 200 cells/well were seeded in six-well plates and cultured for 14 days, then the cells were fixed in methanol and stained with 0.2% crystal violet. The colonies with >50 cells were pictured with ImageScanner (GE, USA) and counted using ImageJ (NIH, MD, USA).

### Wound Healing Assay

Cells infected with lentiviral constructors were detached with trypsin and then seeded with DMEM/F12-10% FBS in a six-well plate in triplicates. After 24 h, the middle cells of each well were scratched with a 10-μl sterile pipette tip to make an empty line *via* washing off the detached cell twice with PBS. After 48 h, the pictures of cells that migrated into the empty line were taken under microscope and analyzed by ImageJ.

### Prediction of Target Genes of SNHG8 and hsa-miR-512-5p

The lncRNASNP2 database (http://bioinfo.life.hust.edu.cn/lncRNASNP#!/) was utilized for the prediction of the binding site in SNHG8 for hsa-miR-512-5p and analyzed with inmiRDB (http://mirdb.org/) and TargetScan (http://www.targetscan.org/vert_72/).

### Dual-Luciferase Reporter, RNA Immunoprecipitation (RIP), and Pull-Down Assays

The wild-type (wt) SNHG8 and mutant (mut) SNHG8 that had the predicted hsa-miR-512-5p-binding site were chemically synthesized by Hanheng Biotechnology Co. Ltd. (Beijing, China) and inserted into pSI-check2 luciferase reporter plasmids (Promega Corporation, Madison, WI, USA) to make the pMIR-SNHG8-wt (SNHG8-wt) and pMIR-SNHG8-mut (SNHG8-mut) reporter plasmids. Similarly, the reporter plasmids, TRIM28-wt and TRIM28-mut, were also made. For the reporter assay, when cells in 24-well plates grew to 70% confluence, the reporter plasmids were co-transfected with hsa-miR-512-5p mimics or miR-NC into the cells using Lipofectamine 2000 reagent. After 48 h, the luciferase activity in the transfected cells was detected with Dual-Luciferase Reporter Assay System (Promega Corporation, Madison, WI, USA) on a Synergy H4 reader. The relative luciferase activity was normalized with Renilla luciferase activity.

For the RIP assay, the EZ-Magna RIP Kit (Sigma, St. Louis, MO, USA) was used. In brief, the lysate of 1 × 107 AGS/BX-1 cells in RIP lysis buffer was incubated with Anti-IgG or Anti-Ago2-coated magnetic beads for 6 h. RNAs of SNHG8 and miR-512-5p enriched on the beads was extracted and quantified by reverse-transcription quantitative polymerase chain reaction (qRT-PCR).

RNA pull-down assay was carried out to assess the interaction of miR-512-5p with SNHG8 using a Magnetic RNA Pull-Down Kit (Thermo Fisher Scientific). The miR-512-5p mimic (Bio-miR-512-5p) and negative control (Bio-miR-NC) were biotinylated by RiboBio Inc. (Guangzhou, China). AGS/BX-1 cells were transfected with 100-nm biotinylated probes for 48 h; then, the cell lysates were incubated with streptavidin-coated magnetic beads overnight. The RNA enriched on the beads was separated, and the content of SNHG8 was detected by qRT-PCR.

### Western Blot Analysis

Cells were harvested and lysed with RIPA buffer (50 mM Tris–Cl, pH 8.0, 150 mM NaCl, 5 mM EDTA, 0.1% SDS, 1% NP-40) containing protease inhibitor cocktail (Abcam, Cat No. ab65621). The lysates were centrifuged at 12,000 rpm for 30 min at 4°C. The protein concentrations of supernatants were determined by BCA protein assay (Thermo Scientific, Rockford, IL, USA). The protein extract of each sample (25 μg) was electrophoresed on 10%–12% polyacrylamide gel with sodium dodecyl sulfate and then transferred onto nitrocellulose membrane (Millipore A) at 100 V for 1.5 h. After being blocked with 3% BSA in TBST (TBS-1% Tween 20) for 1 h, the membranes were incubated with 1:1,000 primary antibodies (purchased from CST, Danvers, MA, USA) of TRIM28 (Cat No. #4124), VIM (Cat No. 5741S), CCND1 (Cat No. 55506s), PCNA (Cat No. D3H8P), Bcl-2 (Cat No. 5071S), CDH1 (Cat No. 3195s), CDH2 (Cat No. 13116s), Snail (Cat No. 3879S), or PARP(Cat No. 46D11) overnight at 4°C, respectively, then washed and further incubated with secondary horseradish peroxidase-conjugated anti-rabbit IgG. Finally, protein bands were detected by developing the blots with Immobilon ECL Ultra Western HRP Substrate (Millipore, Cat No. WBULS0500) and pictured on Image Station 4000MM Pro (Carestream, Canada). ImageJ was used to quantify protein levels relative to load control β-actin.

### Transplantation of Zebrafish With SNHG8 Knockout Gastric Cancer EBV-Positive Cells AGS/BX-1

Transgenic zebrafish TG (apo14-GFP) was provided by the Institute of Hydrobiology, Chinese Academy of Sciences. The culture condition of adult fish was 26.5°C, and the light dark ratio was 12 h:12 h. AGS-BX-1/SNHG8-SH cells and AGS-BX-1/NC cells were injected with a Pico-liter injector. Under a microscope, 1,500 cells were injected into the IVF Development Zone of apo14-EGFP zebrafish yolk sac on the second day after fertilization. After injection, the embryos were cultured in a 33°C incubator and transferred to a 35°C incubator 24 h later. The distribution and expression of red fluorescence in the abdominal cavity of zebra were observed under a laser scanning confocal microscope (LSM 710, Carl Zeiss AG, Oberkochen, Germany). The expression level of red fluorescence in the abdominal cavity was analyzed by ImageJ 1.48v. Imaging and quantification of the results were performed with an inverted SP5 STED confocal microscope (Leica, Germany). At least 40 zebrafishes in each group were analyzed, and three representative images were used. All the experiments were repeated three times.

### Statistical Analysis

All data were processed by SPSS 19.0 software (SPSS Company, Chicago, USA). Pearson correlation analysis was used. The two-tailed *t test* was used to test the difference between groups. *Paired or nonparametric Kruskal–Wallis test* was used to evaluate the relationship between SNHG8 level and other characteristics. The survival curve was calculated by the *Kaplan–Meier* method. A p value less than 0.05 (*p <0.05, **p <0.01, ***p <0.001, and ****p <0.0001) was statistically significant.

## Results

### Expression of EBV-Related Protein BFRF1 in GC and Its Upregulation of the Expressions of SNHG8 and TRIM28

**Figure 1:**
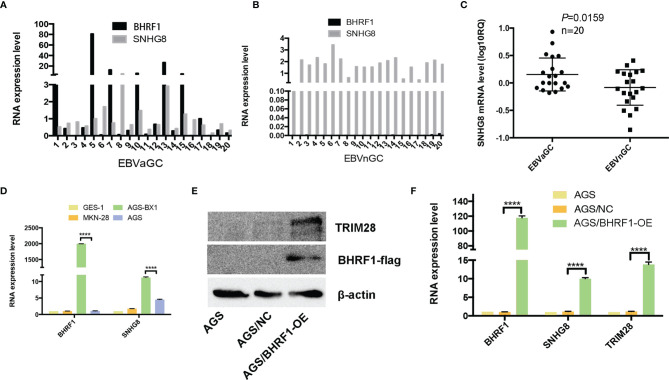
Expression of *BFRF1* in GC and GC cell lines and its regulation with SNHG8 and *TRIM28*. **(A, B)** *BHRF1* was expressed in EBVaGC tissues at different levels, while it was almost not expressed on EBVnGC. **(C)** The SNHG8 expression was much higher in EBVaGC tissues than that in EBVnGC tissues (*p* = 0.0159). RQ = 2^−ΔΔCt^, where Ct values were generated from qPCR. **(D)** The expression of *BHRF1* and SNHG8 was much higher in EBV^+^ AGS-BX cells than in EBV^-^ GC cell lines (AGS, MKN28). **(E)** Overexpression of EBV protein BFRF1 resulted in an increased TRIM28 demonstrated by Western blot analysis. **(F)** Overexpression of EBV protein *BFRF1* enhanced the transcription of SNHG8 and *TRIM28* demonstrated by qRT-PCR. ****p < 0.0001.

BHRF1 (EBV-encoded small ribonucleic acid 1) is an EBV oncogene confirmed recently. **Figures 1A, B** show that BHRF1 was expressed in EBVaGC cases, but not expressed in EBVnGC (EBV-negative GC) tissues. In addition, the BHRF1 expression was significantly higher in AGS-BX (EBV infected GC cell line) than that in non-EBV-infected GC cell lines (AGS and MKN28) (**Figure 1D**). Besides, SNHG8 expression was also much higher in EBVaGC and AGS-BX than in EBVnGC- and in non-EBV-infected GC cell lines (AGS, MKN28) (**Figures 1C, D** **)**.

To determine the relationship between BHRF1 and SNHG8, we constructed a vector pcdna3.1-flag-BHRF1 to stably express human BHRF1 protein in EBV-negative AGS cells. Western blot assays (WB) show that the expression levels of BFRF1 and TRIM28 were much higher in AGS/BHRF1 cells than those in AGS/NC cells (**Figure 1E**). qRT-PCR assays revealed that the expression of BHRF1, SNHG8, and TRIM28 was much higher in the AGS/BHRF1 group than in the AGS/NC group (**Figure 1F**). These results indicated that the over-expression of EBV-related protein BFRF1 in EBV-negative AGS cells upregulated the expression of SNHG8 and TRIM28.

### Association of SNHG8 With Clinicopathological Features and Prognosis of EBVaGC

**Figure 2:**
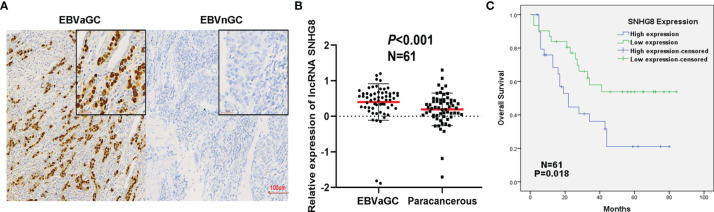
Poor prognosis of EBVaGC was associated with the upregulation of SNHG8. **(A)** EBER in gastric cancer detected by *in situ* hybridization. Left: EBER is positive in EBV-associated gastric cancer (EBVaGC). Right: EBER is negative in non-EBV-associated gastric cancer (EBVnGC). **(B)** The expression of SNHG8 was higher in 61 cases of EBVaGC than that in their paracancerous tissues (*p* < 0.001). **(C)** Kaplan–Meier curves showed that patients with a high level of SNHG8 had a poor OS (*p* = 0.018).

With *in situ* hybridization, EBVaGC was recognized by the expression of EBER1 in nuclei of cancer cells; EBVaGC is a group of lymphoepithelioma-like diffuse-type carcinoma with dense lymphocytic infiltration. As shown in **Figure 2A**, EBVaGC had EBER1-positive nuclei and surrounded with lymphoid stroma.

To determine the expression of SNHG8 in EBVaGC, qRT-PCR assays were performed in 61 cancer tissues and paired paracancerous tissues. SNHG8 was expressed at significantly higher levels in EBVaGC compared to the level in paracancerous tissues (**Figure 2B**). The average log10 relative expression levels in cancer tissues were ~0.41, a two-fold increase compared to that in paracancerous tissues (~0.20). Furthermore, EBVaGC tissue samples were divided into two groups (SNHG8 high- and low-expression groups) using the median mRNA level of SNHG8 as the cutoff value. As shown in **Table 1**, the overexpression of SNHG8 had a significant correlation with tumor size (*p* = 0.036) and vascular tumor thrombus (*p* = 0.024). Moreover, the overexpression of SNHG8 correlated with the poor prognosis in EBVaGC. Patients with the high levels of SNHG8 had significantly shorter overall survival (OS) than those with low SNHG8 expression (*p* = 0.018; **Figure 2C**). The results suggested that the expression of SNHG8 was positively correlated with tumor size and poor prognosis in EBVaGC.

**Table 1:** Relationship of SNHG8 expression with clinicopathologic characteristics in EBVaGC.

Characteristics	Number of patients	SNHG8 expression		*p*-value
		Low	High	
**All patients**	61	30	31	
**Gender**				0.479
Male	49	23	26	
Female	12	7	5	
**Age**				0.252
<60	35	15	20	
≥60	26	15	11	
**Lauren’s type**				0.221
Intestinal type	49	26	23	
Diffuse type	12	4	8	
**Size(cm)**				0.036*
<5	41	24	17	
≥5	20	6	14	
**Vascular tumor thrombus**				0.024*
Absent	45	26	19	
Present	16	4	12	

*p < 0.05.

### Overexpression of SNHG8 Promotes the Proliferation, Migration, and Invasiveness of EBVaGC

**Figure 3:**
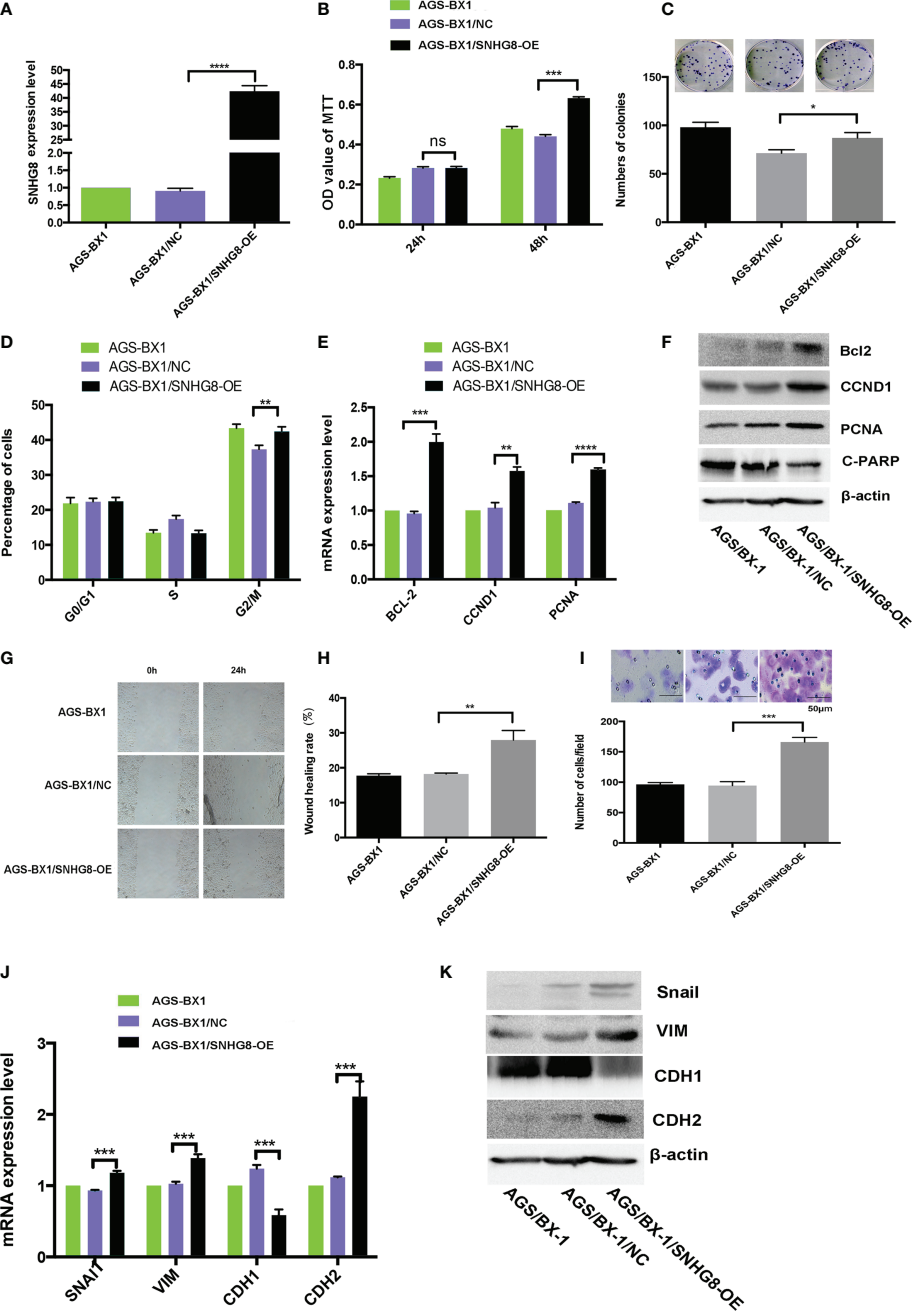
Overexpression of SNHG8 promoted the proliferation, migration, and invasion of EBVaGC. **(A)** AGS-BX1 cells with SNHG8 gene overexpression (AGS-BX1/SNHG8-OE)had a high level of the expression of SNHG8 demonstrated by qPCR as compared to vector alone control (AGS-BX1/NC) and wild-type cells (AGS-BX1). The overexpression of SNHG8 promoted the malignant behaviors of AGS-BX1/SNHG8-OE cells in the following aspects: **(B)** increasing cell proliferation as measured with MTS assay; **(C)** enhancing colony formation; **(D)** stopping cells at G2/M; **(E, F)** increasing the expression of proliferation-related genes of *BCL-2*, *CCND1*, *C-PARP*, and *PCNA*, confirmed by qRT-PCR and Western blot; **(G, H)** promoting the migration measured with the wound healing assay and the invasion measured with the transwell invasive assay; **(I)** and enhancing the expression of invasion/metastasis-related-genes, *Snai1*, *VIM*, CDH1, and *CDH2* as demonstrated by qRT-PCR and Western blot **(J, K)**. *p < 0.05; **p < 0.01; ***p < 0.001; ****p < 0.0001. ns mean not statistical significance.

To assess the effects of SNHG8 on the malignant behaviors of EBVaGC, EBV-positive gastric carcinoma cell lines AGS-BX1 were infected with HBLV-NC or HBLV-SNHG8-OE lentivirus. RT-qPCR analysis revealed that SNHG8 expression was efficiently increased in AGS-BX1/SNHG8-OE as compared with AGS-BX1/NC (**Figure 3A**). The effect of SNHG8 overexpression on the enhanced proliferation and colony formation of AGS-BX1 cells was demonstrated in MTT assay (**Figure 3B**) and colony assay (**Figure 3C**). In addition, SNHG8 overexpression markedly promoted cell cycle arrest at the G2/M phase of AGS-BX1 compared with control cell AGS-BX1/NC (**Figure 3D**). Furthermore, results of RT-qPCR and WB showed that the expression of proliferation-related genes *CCND1*, *PCNA*, and anti-apoptotic *BCL-2*, metastasis-related genes *Snai1VIM* and *CDH2* were upregulated, while *PARP1* and *CDH1* were downregulated in AGS-BX1/SNHG8-OE cells (**Figures 3E, F, J, K** **)**. Besides, the results of wound healing and transwell invasion assays showed that the upregulation of SNHG8 resulted in an increased abilities of wound healing (**Figures 3G, H** **)** and transwell invasiveness (**Figure 3I**). These findings suggested that SNHG8 might exert oncogenic effects on the aggressiveness of EBVaGC cell *in vitro*.

### Silence of SNHG8 Inhibits the Proliferation, Migration, and Invasiveness of EBVaGC

**Figure 4:**
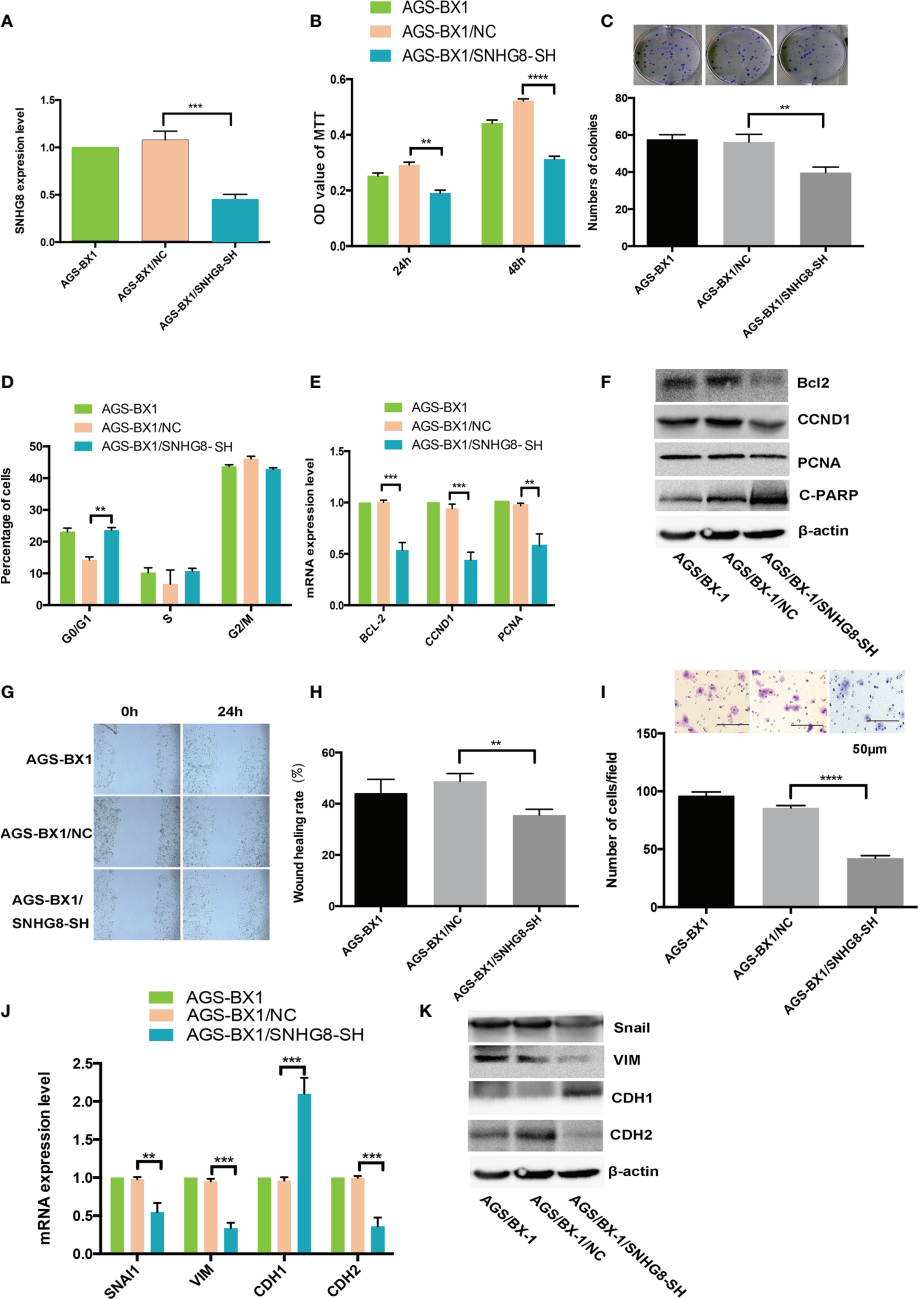
Silencing SNHG8 inhibited the proliferation, migration, and invasion of EBVaGC. **(A)** SNHG8 gene knockdown in AGS-BX1 cells (AGS-BX1/SNHG8-SH) had a low expression of SNHG8 quantified by qPCR as compared to vector alone control (AGS-BX1/NC) and wild-type cells (AGS-BX1). SNHG8 silencing reduced the malignant behaviors of AGS-BX1/SNHG8-SH cells in following aspects: the cell proliferation as measured with MTT assay **(B)**; colony formation **(C)**; cells in G0/1 **(D)**; the expressions of proliferation-related-genes of *BCL-2*, *CCND1*, *C=PARP*, and *PCNA* demonstrated by qRT-PCR and Western blot **(E, F)**; the migration measured with the wound healing assay **(G, H)**; the invasion measured with the transwell invasive assay **(I)**; and the expressions of invasion/metastasis-related-genes of *Snail*, *VIM*, CDH1, and *CDH2* demonstrated by qRT-PCR and Western blotting **(J, K)**. **p < 0.01; ***p < 0.001; ****p < 0.0001.

To assess if downregulation of SNHG8 could reduce the malignant behaviors of EBVaGC, AGS-BX1 was infected with HBLV-NC or HBLV-SNHG8-SH lentivirus. RT-qPCR analysis showed that the SNHG8 expression was significantly reduced in AGS-BX1/SNHG8-SH as compared with AGS-BX1/NC (**Figure 4A**). SNHG8 silencing markedly inhibited the proliferation and colony formation of AGS-BX1 (**Figures 4B, C** **)**. In addition, compared with AGS-BX1/NC, there was a significant amount of cells arrested at the GO/G1 phase in AGS-BX1/SNHG8-SH as measured by a Muse cell analyzer (**Figure 4D**). RT-qPCR and WB results showed that the expressions of proliferation-related genes *BCL-2*, *CCND1*, *PCNA*, and metastasis-related genes *Snai1VIM* and *CDH1* were downregulated, while *PARP1* and *CDH1 were* upregulated in AGS-BX1/SNHG8-SH cells (**Figures 4E, F, J, K** **)**. Furthermore, the wound healing migratory ability and transwell invasive abilities were inhibited after the downregulation of SNHG8 (**Figures 4G–I**). These findings suggested that silencing of SNHG8 inhibited the proliferation, migration, and invasiveness of EBVaGC cells *in vitro*.

### SNHG8 Sponges hsa-miR-512-5p and Regulates *TRIM28*

**Figure 5:**
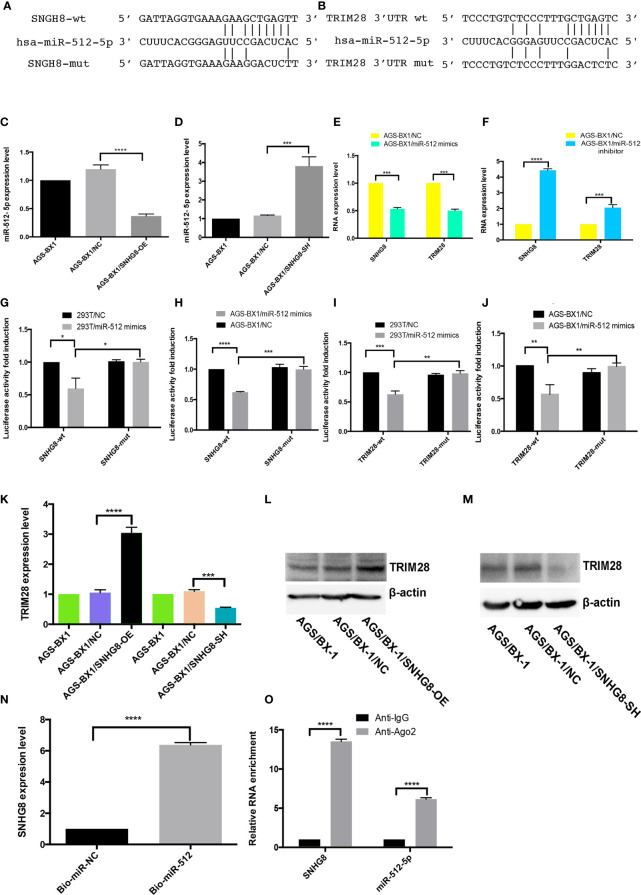
SNHG8 exerted its function through sponging hsa-miR-512-5p and upregulating of TRIM28. **(A, B)** Sequence alignment of hsa-miR-512-5p with the putative binding sites in the wild-type and mutant regions of SNHG8 and TRIM28. **(C, D)** Hsa-miR-512-5p expression was decreased in AGS-BX1/SNHG8-OE cells and increased in AGS-BX1/SNHG8-SH cells tested with qRT-PCR. **(E, F)** The expression of SNHG8 and *TRIM28* was reduced in AGS-BX1/miR-512-5p mimic cells tested with qRT-PCR. **(G–J)** Dual-luciferase reporter assay showed that hsa-miR-512-5p mimics reduced the intensity of fluorescence in HEK293T or AGS-BX1 cells transfected with SNHG8-Wt or TRIM28-Wt, but not in the controls of SNHG8-Mut or TRIM28-Mut vector. **(K–M)** TRIM28 expression was increased in AGS-BX1/SNHG8-OE cells and decreased in AGS-BX1/SNHG8-SH cells tested with qRT-PCR and Western blot analysis. **(N)** SNHG8 expression was measured with qRT-PCR after RNA pull-down assay using Bio-miR-NC and Bio-miR-512-5p. **(O)** SNHG8 and miR-512-5p levels were determined by qRT-PCR after Ago 2 or IgG RIP assay. *p < 0.05; **p < 0.01; ***p < 0.001; ****p < 0.0001.

Potential miRNAs associated with SNHG8 in EBVaGC cells were examined since lncRNAs mainly functioned as miRNA spongers. Analyzed with the lncRNASNP2 database, hsa-miR-512-5p turned out to be a possible target of SNHG8. Further analyzed with the TargetScan and miRDB databases, TRIM28 turned out to be a possible target of hsa-miR-512-5p. Putative binding sites of hsa-miR-512-5p and wild-type regions of SNHG8 and *TRIM28* are shown in **Figures 5A, B**. RT-qPCR analysis showed that the overexpression or silencing of SNHG8 significantly decreased or increased hsa-miR-512-5p expression (**Figures 5C, D** **)**, respectively. In parallel, the overexpression of hsa-miR-512-5p decreased the functions of SNGH8 and *TRIM28* expression, knockdown of hsa-miR-512-5p increased the functions of SNGH8 and *TRIM28* expression (**Figures 5E, F** **)**.

To explore whether SNHG8 and *TRIM28* were functional targets of hsa-miR-512-5p, dual-luciferase reporter assay was performed. The luciferase activity was significantly reduced when hsa-miR-512-5p mimics were added into cultured SNHG8-Wt or TRIM28-Wt-co-transfected HEK293T and AGS-BX1 cells, while there was no effect on the same cell transfected with SNHG8-Mut or TRIM28-Mut vector (**Figures 5G–J** **)**.

As expected, knocking down of SNHG8 with lentivirus significantly suppressed the expression of *TRIM28* in AGS-BX1 cells (**Figures 5K, M** **)**. In contrast, the overexpression of SNHG8 significantly increased the expression of *TRIM28* in AGS-BX1 cells (**Figures 5K, L** **)**. RNA pull-down analysis presented that SNHG8 could bind to hsa-miR-512-5p (**Figure 5N**). In addition, the result of the NA immunoprecipitation (RIP) assay showed that SNHG8 and hsa-miR-512-5p could be co-enriched in an Ago2-dependent manner (**Figure 5O**).

Taken together, these results demonstrated that SNHG8 and TRIM28’s 3 ′UTR had hsa-miR-512-5p-binding sites. Hsa-miR-512-5p was an inhibitor of SNHG8 and a blocker of TRIM28 in the EBVaGC progression.

### Enhancement of *TRIM28* Is Critical for SNHG8-Mediated Malignant Behaviors

**Figure 6:**
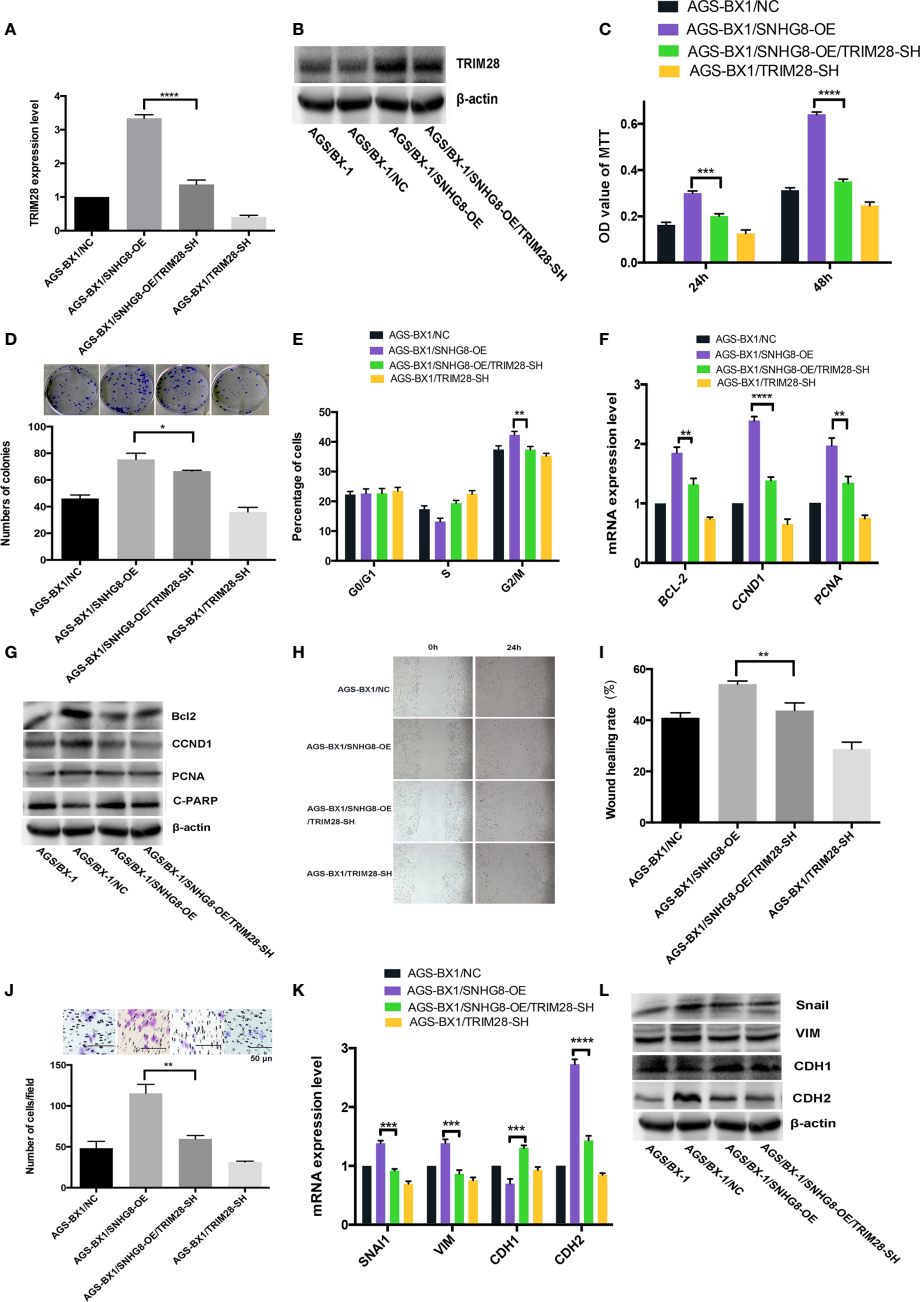
Enhancing of *TRIM28* was critical for SNHG8-mediated malignant behaviors. **(A, B)** *TRIM28* expression was increased in AGS-BX1/SNHG8-OE cells and reduced by silencing of *TRIM28* tested by RT- PCR and Western blot. **(C)** SNHG8 enhanced cell proliferation could be reduced by silencing of *TRIM28* tested with MTS assay. **(D)** SNHG8 enhanced colony formation could be reduced by silencing of *TRIM28*. **(E)** SNHG8-enhanced cell cycle stopping at G2/M could be reduced by silencing of *TRIM28* as detected by flow cytometry. **(F, G)** SNHG8-enhanced expression of *BCL-2*, *CCND1*, *C-PARP*, and *PCNA* could be reduced by silencing of *TRIM28* tested with qRT-PCR and Western Blot. **(H, I)** SNHG8-enhanced cell migration could be reduced by silencing of *TRIM28* tested with wound healing assay. **(J)** SNHG8-enhanced cell invasion could be reduced by silencing of *TRIM28* tested with transwell assay. **(K, L)** SNHG8-enhanced expressions of invasion/metastasis-related-genes of *VIM* and *CDH2* could be reduced by silencing of *TRIM28* tested with qRT-PCR and Western blot. *p < 0.05; **p < 0.01; ***p < 0.001; ****p < 0.0001.

To test our hypothesis that *TRIM28* might contribute to SNHG8-mediated malignant behaviors, the effects of SNHG8 on the *TRIM28* expression in AGS-BX1 cells were studied. A rescue assay by silencing *TRIM28* in AGS-BX1/SNHG8-OE was performed. Results of qPCR and WB revealed that the expression level of *TRIM28* in AGS-BX1/SNHG8-OE/TRIM28-SH was significantly lower than that in AGS-BX1/SNHG8-OE (**Figures 6A, B** **)**. Silencing of *TRIM28* reduced the proliferation and colony formation of AGS-BX1/SNHG8-OE cell as determined by MTT assay (**Figure 6C**) and colony assay (**Figure 6D**). The silencing *TRIM28* markedly reduced the cell cycle arrest at the G2/M phase of AGS-BX1 compared with AGS-BX1/SNHG8-OE (**Figure 6E**). In addition, results of qRT-PCR and WB assay showed that the expression of a panel of proliferation or apoptosis-related genes, such as *BCL-2*, *CCND1*, and *PCNA*, was reduced after *TRIM28* was silenced in AGS-BX1/SNHG8-OE cells (**Figures 6F, G** **)**. Furthermore, the migratory and invasive abilities of AGS-BX1/SNHG8-OE cell lines greatly reduced after the silencing of *TRIM28*, as demonstrated in wound healing (**Figures 6H, I** **)** and transwell invasion assays (**Figure 6J**). The expressions of downstream molecules (*Snai1*, *VIM*, and *CDH2*) related with metastasis were downregulated as determined by qRT-PCR and WB, while CDH1 was upregulated silencing TRIM28 in AGS-BX1/SNHG8-OE cells (**Figures 6K, L** **)**.

These findings suggest that the enhancement of *TRIM28* is critical for SNHG8-mediated malignant behaviors in EBVaGC cells *in vitro*.

### SNHG8 Promotes Growth of EBVaGC Tumor in Zebrafish

**Figure 7:**
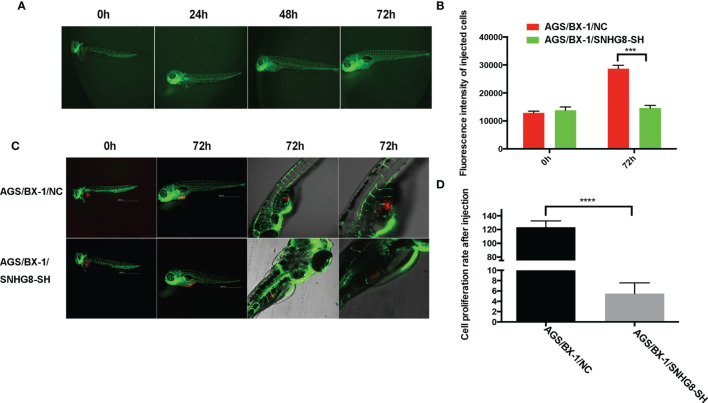
SNHG8 promoted the growth of EBVaGC tumor in zebrafish. **(A)** The growth of zebrafish was not affected by the injection of AGS-BX1 cells in 3 days. **(B)** Representative images of zebrafish from the control (AGS-BX1-NC) and AGS-BX1-SNHG8-SH groups with zoom-in of cancer cells. **(C, D)** Analysis of cancer cell numbers of each group in zebrafish assay. AGS-BX1 cells transfected with red florescence gene (AGS-BX1-NC, AGS-BX1-SNHG8-SH) could form tumor in zebrafish, which could be reduced by silencing of SNHG8 as determined by red fluorescence integral density and cancer cells in zebrafish abdominal cavity. ***p < 0.001; ****p < 0.0001.

To explore the role of *SNHG8* in tumor growth of EBVaGC *in vivo*, the zebrafish EBVaGC tumor xenograft model was used. Injection of AGS-BX1 cells did not affect the normal development of zebrafish (**Figure 7A**). However, the growth of SNHG8-knocked-down mCherry-expressing AGS-BX1 cells significantly decreased compared to the vector-alone control group on 3 days after injection of the same number of cells into the circulation of zebrafish embryos, as demonstrated by the low intensity of red fluorescence (**Figures 7B, C** **)** and low proliferation rate (**Figure 7D**) in the AGS/BX-1/SNHG8-SH group.

Taken together, these data show that SNHG8 could sponge hsa-miR-512-5p and regulate the expression of *TRIM28* in EBVaGC to enhance malignant behaviors.

## Discussion

In this study, several facts were revealed for the first time for EBVaGC, a distinct subtype of GC: (**1**) the existence of BHRF1 was correlated with the expression of SNHG8; (**2**) BHRF1^+^/SNHG8 ^high^ had a poor prognosis; (**3**) there was a coexistence of BHRF1^+^/SNHG8 ^high^/miR-512-5p ^low^/TRIM28^high^ in EBVaGC; (**4**) the chain reaction was that *BHRF1* triggered the high expression of SNHG8, which sponged hsa-miR-512-5p and upregulated *TRIM28*; (**5**) the malignant behaviors of BHRF1^+^/SNHG8 ^high^/miR-512-5p ^low^/TRIM28^high^ EBVaGC were exerted by a set of proliferation-related genes, such as *BCL-2*, *CCND1*, *PCNA*, and *PARP1* and metastasis-related genes, such as *Snail*, *VIM*, *CDH1*, and *CDH2*. This study further explored the molecular mechanism of our previous finding that BHRF1-SNHG8 was a key regulator of EBVaGC (21).

EBV is an oncogenic virus, associated with tumors from epithelia and hematopoietic cells (2, 25). Most studies have focused on genomes of EBV, such as EBER, EBNA-1, and BART (2, 26, 27), while this study further studied the role of *BHRF1* in the development and progression of EBVaGC. The upregulation of *BHRF1* resulted in over-expression of SNHG8, connecting the viral component to the human oncogene, possibly leading to this unique subtype of GC.

Cellular lncRNAs can be differentially induced by EBV infection. The dysregulated lncRNAs probably modulate tumorigenesis and other biological functions (28–30). Our result is consistent with others’ reports that SNHG8 is associated with the progression in multiple types of cancer in the liver, colon, lung, ovary, prostate, esophagus, and so on (15, 31–35). Our data support that the acting mechanism of SNHG8 could be multiple: (**1**) it activated *BCL-2*, preventing GC cells from apoptosis; (**2**) it activated *CCND1*, regulating the cell cycle; and (**3**) it activated *CDH1*, *CDH2Snai1*, and *VIM*, enhancing the epithelial–mesenchymal transition, thus promoting the migration, invasion, and metastasis of GC cells.

LncRNAs are involved in the regulation of gene transcription, post-transcription, and epigenetic modulation (12, 36). Since lncRNAs and mRNAs share miRNAs’ response elements, they compete for binding to these miRNAs, regulating the expression of each other. The interaction between these RNA molecules forms a network of complex posttranscriptional regulation (21, 37–40). Bioinformatics analyses revealed that miR-512-5p shared common binding sites with SNHG8 and *TRIM28*. Our dual-luciferase reporter assay indeed confirmed that SNHG8 functions as a ceRNA in the miR-512-5p/TRIM28 axis as SNHG8 bound to miR-512-5p, while *TRIM28* bound to miR-152-5p. Furthermore, subsequent biotinylated miRNA pull-down assays confirmed the competitive binding between SNHG8 and *TRIM28* to miR-512-5p. Moreover, the rescue experiment showed that SNHG8 significantly reduced the effects of miR-512-5p on *TRIM28*.

*TRIM28*, a member of a conserved family of transcription co-factors, has diverse functions for the regulation of cell proliferation, DNA repair, and differentiation (40–43). *TRIM28* regulated E-cadherin and N-cadherin, resulting in EMT in lung cancer cells (44). *TRIM28* prevented *TRIM24* from SPOP-mediated degradation, promoting the progression of prostate cancer (42). *TRIM28* also affected the mTOR signaling pathway, resulting in the growth of cervical cancer (45). Reduction of *TRIM28* could reduce the malignant behaviors triggered by BHRF1-SNHG8.

Taken together, our *in vitro* and *in vivo* data showed that *BHRF1* upregulated SNHG8, which sponged miR-512-5p, leading to a high level of *TRIM28*, carrying out the malignant behaviors. On the other hand, the silencing of SNHG8 would lead to miR-512-5p upregulation and reduction of TRIM28. This process of BHRF1^+^/SNHG8 ^high^/miR-512-5p ^low^/TRIM28^high^ in EBVaGC directly enhances the activation of a set of tumor-promoting factors, such as proliferation-related genes (*BCL-2*, *CCND1*, *PCNA*, *PARP1*) and metastasis-related genes (*Snail*, *VIM*, *CDH1*, and *CDH2*).

Therefore, silencing of SNHG8 would greatly reduce the malignant behaviors of EBVaGC, which might pave a new road to overcome EBVaGC.

## Conclusion

Our findings demonstrated that *BHRF1* triggered the expression of SNHG8, which sponged miR-512-5p and upregulated *TRIM28* and a set of effectors (such as *BCL-2*, *CCND1*, PCNA, *CDH1*, *CDH2*, *Snail*, and *VIM*) to promote the EBVaGC tumorigenesis and invasion. SNHG8 could be an independent prognostic factor for EBVaGC and sever as target for the therapy.

## Data Availability Statement

The original contributions presented in the study are included in the article/supplementary material. Further inquiries can be directed to the corresponding authors.

## Ethics Statement

The studies involving human participants were reviewed and approved by the Ethics Committee of the Fujian University Cancer Hospital, Fujian Cancer Hospital (Fuzhou, China). The patients/participants provided their written informed consent to participate in this study.

## Author Contributions

XGL and TH designed the research. CZ, JL, DH, HL, and KL performed the experiments. YS, XGL, LZ, and TH analyzed the data. DH, XZ, LZ, and XDL conducted the histological/pathological analysis. XDL, JL, and CZ wrote the paper. LZ and TH edited the paper. All authors contributed to the article and approved the submitted version.

## Funding

This study was supported by the Natural Science Foundation of Fujian Province (No. 2019J01196, 2020J011109), Fujian Provincial Clinlical Research Center for Cancer Radiotherapy and Immunotherapy (NO. 2020Y2012), the Joint Funds for the Innovation of Science and Technology, Fujian province (No. 2018Y9113), Medical Innovation Program of Fujian Province (No. 2019-CX-5), Strategic Priority Research Program of Chinese Academy of Sciences (XDB38050200, XDA26040304), National Key R&D Program of China (2018YFC0910403, 2017YFC1201200), and Shanghai Municipal Science and Technology Major Project (2017SHZDZX01).

## Conflict of Interest

The authors declare that the research was conducted in the absence of any commercial or financial relationships that could be construed as a potential conflict of interest.

## Publisher’s Note

All claims expressed in this article are solely those of the authors and do not necessarily represent those of their affiliated organizations, or those of the publisher, the editors and the reviewers. Any product that may be evaluated in this article, or claim that may be made by its manufacturer, is not guaranteed or endorsed by the publisher.

## Abbreviations

EBV, Epstein–Barr virus; GC, gastric carcinoma; EBVaGC, EBV-positive GC; EBVnGC, non-EBV-infected gastric cancer; RIP, RNA immunoprecipitation; OS, overall survival; ISH, *in situ* hybridization; EMT, epithelial–mesenchymal transition; TCGA, The Cancer Genome Atlas; SNHG8, small nucleolar RNA host gene 8; BHRF1, EBV-encoded small ribonucleic acid 1; TRIM28, tripartite motif-containing 28; RIP, RNA immunoprecipitation; EBER-1, Epstein–Barr encoding region 1; FFPE, formalin-fixed and paraffin-embedded; MTS, (4,5-dimethylthiazol)-3-(4-sulfophenyl)tetrazolium, inner salt; HBLV-SNHG8, pHBLV-CMV-mcs-3flag-EF1-ZsGReen-T2A-PURO inserted with SNHG8 gene; AGS-BX1/NC, empty vector of AGS-BX1 cell infection; AGS-BX1/SNHG8-OE, SNHG8 overexpression AGS-BX1 cell; AGS-BX1/SNHG8-SH, SNHG8 knockdown AGS-BX1 cell; AGS/BHRF1-OE, BHRF1 overexpression AGS cell; SNHG8-wt, SNHG8/miR-512-5p-binding site wild-type cells; SNHG8-mut, SNHG8/miR-512-5p-binding site mutant cells; TRIM28-wt, TRIM28/miR-512-5p-binding site wild-type cells; TRIM28-mut, TRIM28/miR-512-5p-binding site mutant cells; AGS-BX1/SNHG8-OE/TRIM28-SH, SNHG8 overexpression/TRIM28 knockdown AGS-BX1 cell.
